# Triterpenoids of Marine Origin as Anti-Cancer Agents

**DOI:** 10.3390/molecules18077886

**Published:** 2013-07-04

**Authors:** Yong-Xin Li, S. W. A. Himaya, Se-Kwon Kim

**Affiliations:** 1 Marine Bioprocess Research Center, Pukyong National University, Busan 608-737, Korea; E-Mail: lyxycg@hotmail.com; 2 Department of Chemistry, Pukyong National University, Busan 608-737, Korea; E-Mail: himayaswa@yahoo.com

**Keywords:** triterpenoids, anti-cancer, cytotoxic, pharmacological agents, marine origin

## Abstract

Triterpenoids are the most abundant secondary metabolites present in marine organisms, such as marine sponges, sea cucumbers, marine algae and marine-derived fungi. A large number of triterpenoids are known to exhibit cytotoxicity against a variety of tumor cells, as well as anticancer efficacy in preclinical animal models. In this review efforts have been taken to review the structural features and the potential use of triterpenoids of marine origin to be used in the pharmaceutical industry as potential anti-cancer drug leads.

## 1. Introduction

Recently, the search for novel bioactive compounds as anti-cancer agents from marine resources has gained much attention. Triterpenoids are terpenoid derivatives of natural products containing about thirty carbon atoms, and their structures are considered to be derived from the acyclic precursor squalene [1,2]. Triterpenoids are the most abundant secondary metabolite present in marine sources, such as marine sponges [3,4], sea cucumbers [5], marine algae [6], and marine-derived fungi [7]. During a last few years, a great number of biologically active triterpenoids was found to have cytotoxicity against a variety of tumor cells [8,9]. More than 20,000 triterpenoids has been isolated and identified from Nature, which belong to different chemical groups such as squalene, lanostane, dammarane, lupane, oleanane, ursane, hopane, *etc*. [10,11]. This review summarizes the anti-cancer triterpenoids isolated from marine sponges, sea cucumbers, marine algae, and marine fungi that includes isomalabaricane-type triterpenoids (stellettins, stelliferins, and geoditins), polyether triterpenes (sodwanones, raspacionins, sipholenols, sipholenones, and siphonellinols), triterpenoid glycosides (saponins), and tetracyclic triterpenoids and their potential anti-cancer activity. Therefore, this review brings insights to marine triterpenoids as potential candidates to be developed as pharmaceuticals against tumor progression.

An ideal anticancer agent is expected to inhibit, delay or reverse the progression of cancer through its cytotoxicity or apoptosis-inducing properties [12]. The discovery and development of anticancer drugs, especially cytotoxic agents, differ significantly from the drug development process for any other indications. Identification of cytotoxic compounds led the development of anticancer therapeutics for several decades. Cytotoxic drugs are primarily used as anticancer drugs because they are toxic to cancer cells. These drugs have been associated with human cancers at high (therapeutic) levels of exposure and are carcinogens and teratogens in many animal species. Cytotoxic drugs have an effect of preventing the rapid growth and division (mitosis) of cancer cells [13]. During a last few years, great numbers of biologically active triterpenoids are found to have cytotoxicity against a variety of tumor cells. Triterpenoids are highly multifunctional and the antitumor activity of these compounds is measured by their ability to block nuclear factor-kappaB activation, induce apoptosis, inhibit signal transducer, and activate transcription and angiogenesis [14]. Advances in cancer treatment, however, continued to be challenged by the identification of unique biochemical aspects of malignancies that could be exploited to selectively target tumor cells. However, selective elimination of tumor cells using cytotoxic agents is universally applicable approach of cancer treatment. This review will highlight the enormous potential of triterpenoids identified from marine resources as cytotoxic agents against tumor progression.

## 2. Triterpenoids from Marine Sponges

**Figure 1:**
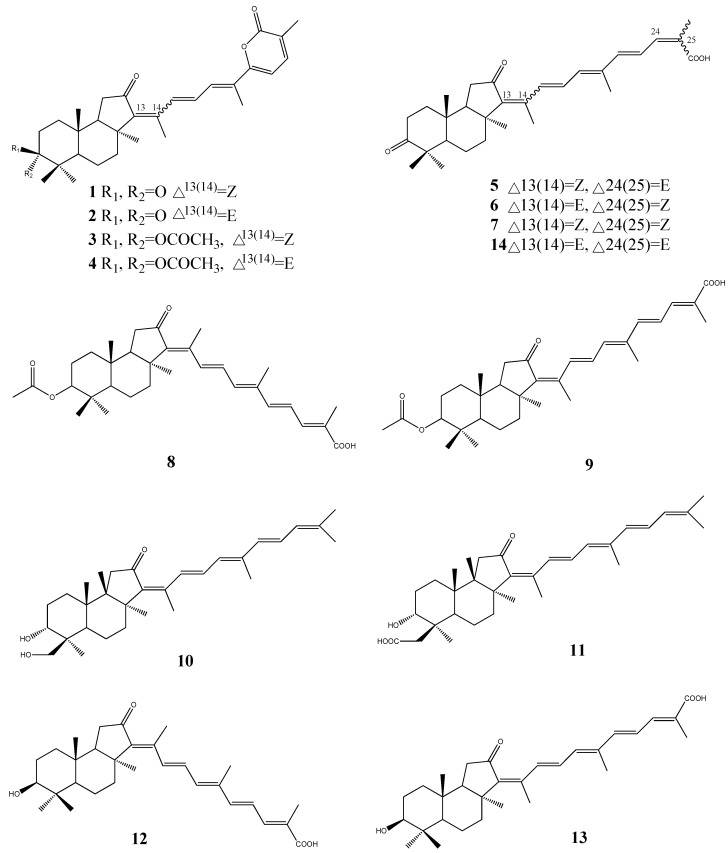
Isomalabaricane-type triterpenoids stellettins from marine sponge.

Isomalabaricane-type triterpenoids are a rare group of triterpenoids with unique skeletons, often found in marine sponges. The cytotoxic isomalabaricane-type triterpenoids stellettins A-K (**1**–**13**, Figure 1) have been reported from the marine sponge species of the genus *Jaspis* [15], *Stelleta* [16,17,18], and *Rhabdastrella* [19]. Stellettin A (**1**) and B (**2**), were isolated from the sponge *Stelletta tmuis* collected from Hainan Island, China in 1994. Stellettin A was significantly toxic to P388 leukemia cells, exhibiting an ED_50_ value of 0.001 µg/mL [20]. Furthermore, Liu *et al*. have demonstrated that stellettin A and stellettin B induce cytotoxicity in HL-60 cells treated for 24 h at 3 µM concentration [21]. The cytotoxic isomalabaricane triterpenoids stellettins A-G (**1**–**7**) have been examined at the National Cancer Institute (Australia) against 60 cell lines. Stelletin C (**3**) and D (**4**) were the most potent derivatives with a mean panel GI_50_ of 0.09 µM. The stelletin E (**5**) and F (**6**) pair was approximately 10-times less potent (mean GI_50_ of 0.98 µM) [16,22].

The isomalabaricane triterpenes, stellettins A-D (**1**–**4**), stellettin H (**8**), stellettin I (**9**) along with rhabdastrellic acid-A (**14**), have been isolated from the marine sponge *Rhabdastrella globostellata*, collected from the Philippines. These compounds have shown selective cytotoxicity towards p21^WAF1/Cip1^-deficient human colon tumor (HCT-116) cells [23].

The cytotoxic isomalabaricane triterpenoids stelletin J (**10**) and K (**11**) from *Rhabdastrella globostellata* have shown activity in an assay measuring stabilization of the binding of DNA with DNA polymerase β. However, stelletin J (**10**) and K (**11**) displayed varying levels of activity toward the A2780 ovarian cancer cell line, revealing structure-based effects on both the level of cytotoxicity and DNA-polymerase β binding [24].

Stelletin L (**12**) and M (**13**) were isolated from the marine sponge* Stelletta tenuis* collected in the South China Sea and both compounds exhibited significant cytotoxic activity against stomach cancer cells (AGS) *in vitro* [18].

**Figure 2:**
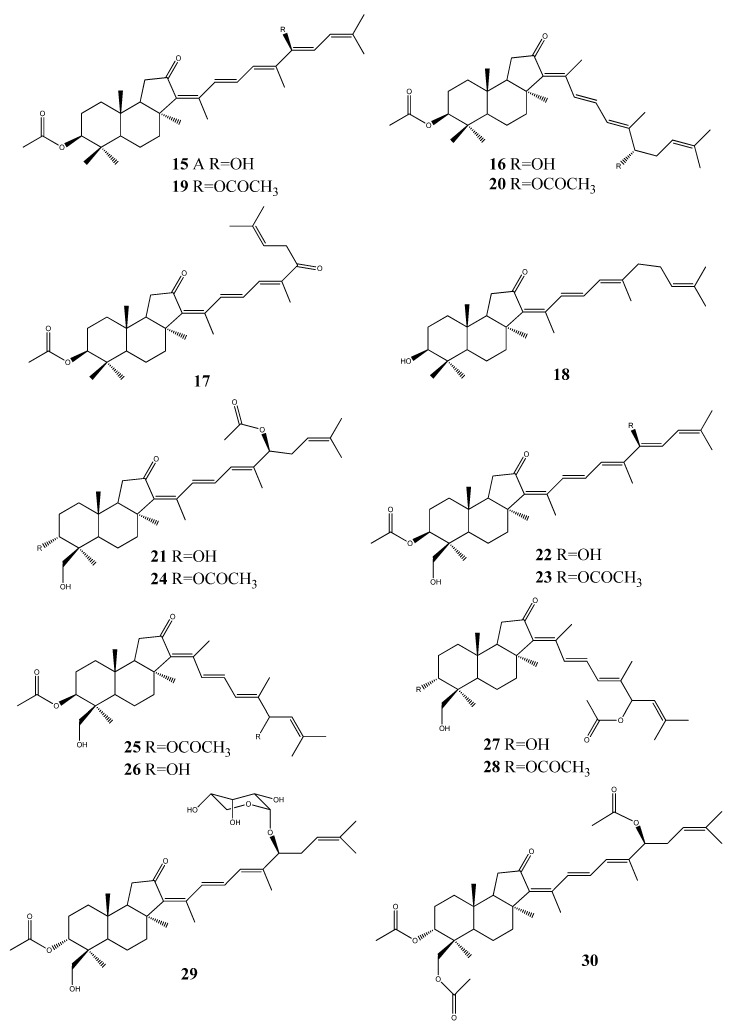
Triterpenoid stelliferins from marine sponges.

Stelliferins A–F (**15**–**20**, Figure 2), antineoplastic isomalabaricane triterpenes were isolated from the Okinawan marine sponge *Jaspis stellifera* [25]. The isomalabaricane triterpenes, stelliferin G (**21**), 29-hydroxystelliferin A (**22**), 29-hydroxystelliferin E (**23**) together with the known triterpenes 3-epi-29-hydroxystelliferin E (**24**), 13(*E*)-29-hydroxystelliferin E (**25**), 29-hydroxystelliferin B (**26**), 13(*E*)-stelliferin G (**27**), and 13(*E*)-3-*epi*-29-hydroxystelliferin E (**28**), were isolated from the organic extract of the sponge *Jaspis sp*. collected in the South Pacific ocean. All compounds were tested against melanoma (MALME-3M) and leukemia (MOLT-4) cells. The mixtures of 29-hydroxystelliferin B (**26**) and 13E-stelliferin G (**27**) have shown highest growth-inhibitory [(IC_50_) 0.11, 0.23, µg/mL, respectively)] activities against MALME-3M [26].

Moreover, the isomalabaricane triterpenoids stelliferin riboside (**29**) and 3-*epi*-29-acetoxystelliferin E (**30**) were isolated from an extract of the sponge *Rhabdastrella globostellata* which was active in an assay measuring stabilization of the binding of DNA with DNA polymerase β. Both compounds have shown to induce 29% and 23% binding, respectively [24].

Four isomalabaricane triterpenes, geoditin A (**31**), geoditin B (**32**), isogeoditin A (**33**), and isogeoditin B (**34**) were isolated from marine sponge *Rhabdastrella aff. distincta*. All compounds were tested against a small panel of human tumor cell lines [19]. Geoditin A (**31**) and geoditin B (**32**) have also been isolated from marine sponge *Geodia japonica*. Geoditin A was the most cytotoxic to HL60 cells [IC 50 Z3 mg/mL (<6.6 mM)], and geoditin B exhibited relatively weak cytotoxicity [27].

**Figure 3:**
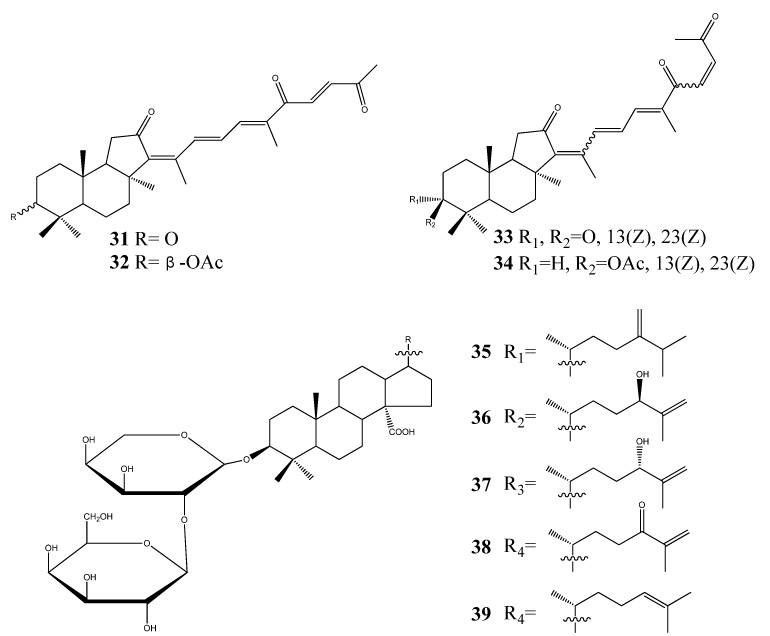
Triterpenoid geoditins from marine sponges.

Five cytotoxic triterpene glycosides, erylosides F1-F4 (**35**–**38**), and erylosides F (**39**) (Figure 3) were isolated from the sponge *Erylus formosus* collected from the Mexican Gulf (Puerto Morelos, Mexico). Four compounds induced the early apoptosis of Ehrlich carcinoma cells, where erylosides F3 have shown the highest activity at a concentration of 100 µg/mL [28].

The special group of triterpenoids named sodwanones: sodwanones A-I (**40**–**48**) and sodwanones K-W (**49**–**61**), have been isolated from the Indo-Pacific sponge *Axinella wltneri* [29]. Sodwanones G (**46**), H (**47**), and I (**48**) have been found to have cytotoxic activity. The compounds have shown cytotoxicity activity against cell cultures of P-388 murine leukemia, A-549 human lung carcinoma, HT-29 human colon carcinoma, and MEL-28 human melanoma. Sodwanones G (**46**), H (**47**), I (**48**) showed high specificity towards human lung carcinoma cell line A-549, where the specificity of sodwanone G was prominent (**46**) [30]. The cytotoxic triterpenes sodwanones K (**49**), L (**50**), and M (**51**) were found to be cytotoxic to P-388 murine leukemia cells [31]. The biological activity of sodwanone S (**57**) was evaluated against 13 human tumor cell lines [32]. Sodwanone V (**60**) inhibited both hypoxia-induced and iron chelator (1,10-phenanthroline)-induced HIF-1 activation in T47D breast tumor cells (IC_50_ 15 µM), and sodwanone V (**60**) was the only sodwanone that inhibited HIF-1 activation in PC-3 prostate tumor cells (IC_50_ 15 µM).

**Figure 4:**
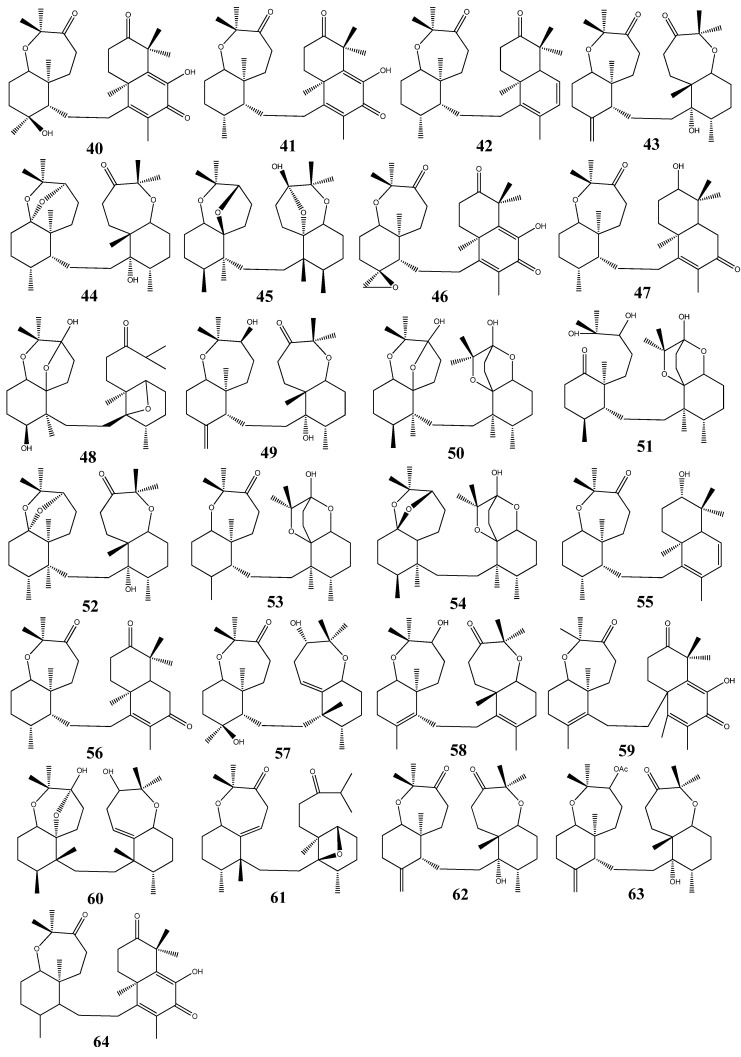
Triterpenoid sodwanones from marine sponges.

Sodwanone A (**40**) and sodwanone T (**58**) inhibited hypoxia-induced HIF-1 activation in T47D cells (IC_50_ values 20–25 µM), and sodwanone V (**60**) showed cytotoxicity to MDA-MB-231 breast tumor cells (IC_50_ 23 µM). Sodwanone derived compounds, 3-*epi*-sodwanone K (**62**), 3-*epi*-sodwanone K 3-acetate (**63**), 10,11-dihydrosodwanone B (**64**) have been isolated from *Axinella sp*., and **62** and **64** also inhibited hypoxia-induced HIF-1 activation in T47D cells (IC_50_ values 20–25 µM) and **63** was cytotoxic to T47D cells (IC_50_ 22 µM) [33] (Figure 4).

**Figure 5:**
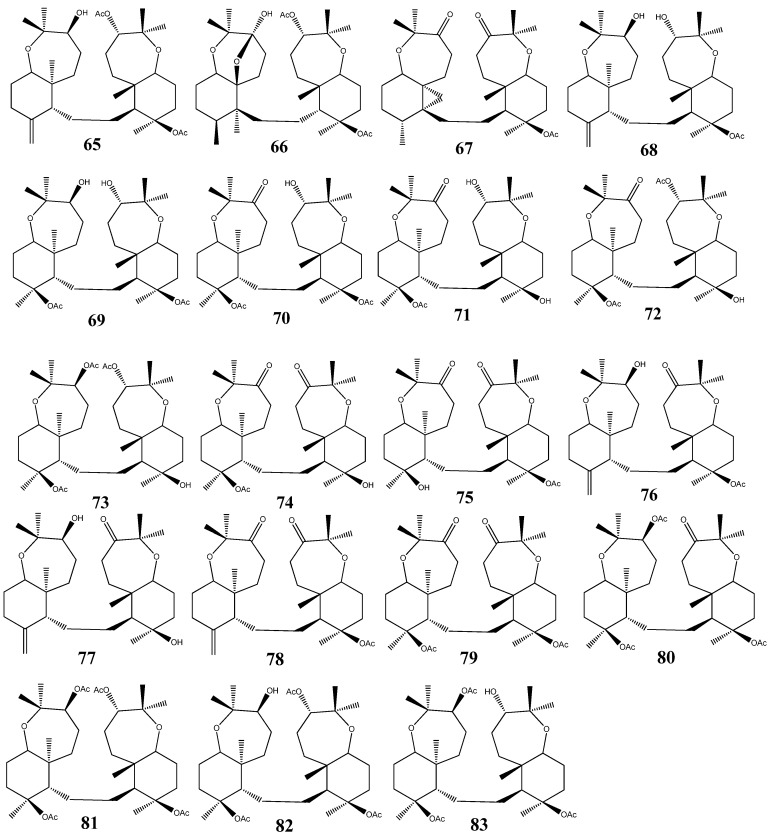
Triterpenoid raspacionins from marine sponges.

Raspacionin triterpinoids **65**–**83** (Figure 5), namely raspacionin (**65**), raspacionins A (**66**), raspacionins B (**67**), 21-deacetylraspacionin (**68**), 10-acetoxy-21-deacetyl-28-hydroraspacionin (**69**), 10-acetoxy-21-deacetyl-4-oxo-28-hydroraspacionin (**70**), 10-acetoxy-15,21-dideacetyl-4-oxo-28-hydroraspacionin (**71**), 10-acetoxy-15-deacetyl-4-oxo-28-hydroraspacionin (**72**), 10-acetoxy-4-acetyl-15-deacetyl-28-hydroraspacionin (**73**), 10-acetoxy-15-deacetyl-4-21-dioxo-28-hydroraspacionin (**74**), 10-hydroxy-4,21-dioxo-28-hydroraspacionin (**75**), 21-oxoraspacionin (**76**), 15-deacetyl-21-dioxo-raspacionin (**77**), 4,21-dioxo-raspacionin (**78**), 10-acetoxy-4,21-dioxo-28-hydroraspacionin (**79**), 10-acetoxy-4-acetyl-21-oxo-28-hydroraspacionin (**80**), 10-acetoxy-4-acetyl-28-hydroraspacionin (**81**), 10-acetoxy-28-hydroraspacionin (**82**) and 10-acetoxy-21-deacetyl-4-acetyl-28-hydroraspacionin (**83**), have been isolated from red sponge, *Raspaciona aculeuta Johnston* (family *Raspailiidae*), and from the Mediterranean sponge *Raspaciona aculeata*. All the compounds have showed cytotoxicity against MCF-7 tumor cell line with IC_50_ values between 4 and 8 µM [34,35,36].

**Figure 6:**
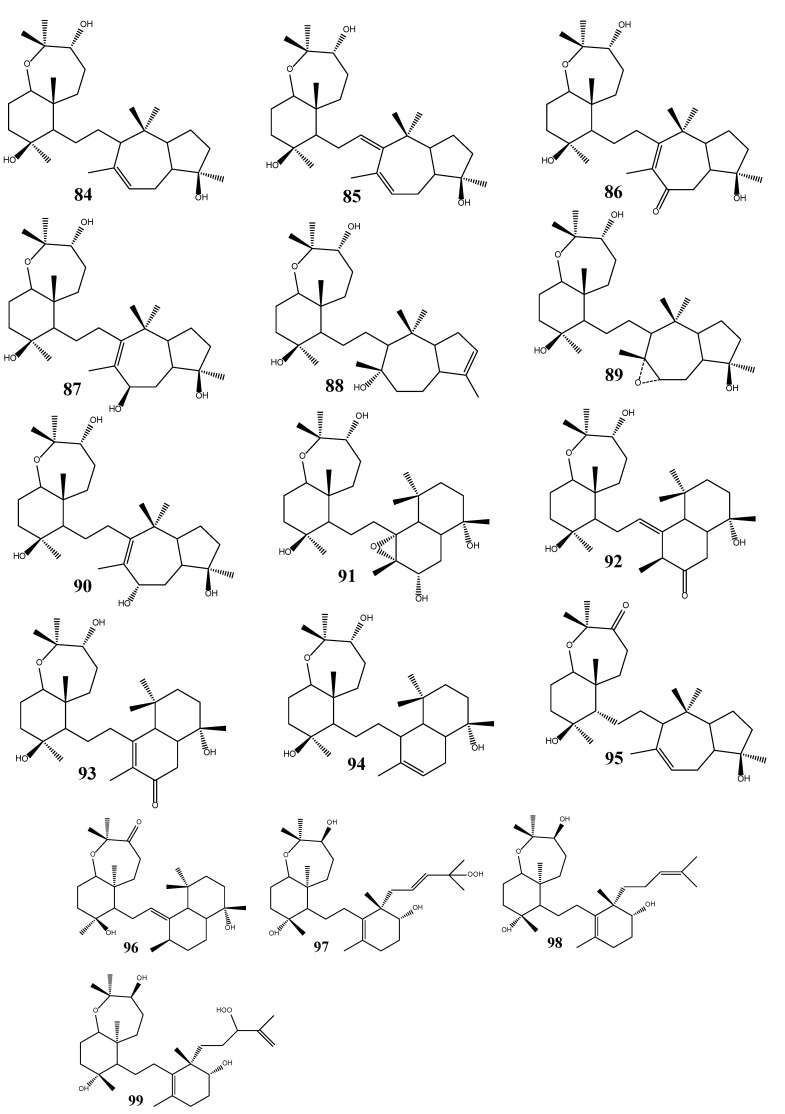
Triterpenoid sipholenols, sipholenones and siphonellinols from marine sponges.

The Red Sea sponge *Siphonochalina siphonella* is a rich source of sipholane triterpenoids including sipholenols (A, C-L) (**84**, **85**–**94**), sipholenones (A, E) (**95**, **96**), and siphonellinols (C, D, E) (**97**, **98**, **99**). Sipholenol A (**84**) and sipholenone A (sipholenol B) are the major sipholane triterpenoids [37]. Sipholenol A was found to have increased the sensitivity of resistant KB-C2 cells [38]. Sipholenol A (**84**), sipholenol I (**91**), sipholenol L (**94**), sipholenone A (**95**), sipholenone E (**96**), siphonellinol C (**97**), and siphonellinol D (**98**) have found to show potent reversal of multidrug resistance in cancer cells that over expressed P-glycoprotein. These compounds enhanced the cytotoxicity of several P-glycoprotein substrate anticancer drugs, and significantly reversed the multidrug resistance phenotype in P-glycoprotein-overexpressing multidrug resistant cancer cells KB-C2 and KB-V1 in a dose-dependent manner [39,40] (Figure 6).

## 3. Triterpenoids from Sea Cucumbers

**Figure 7:**
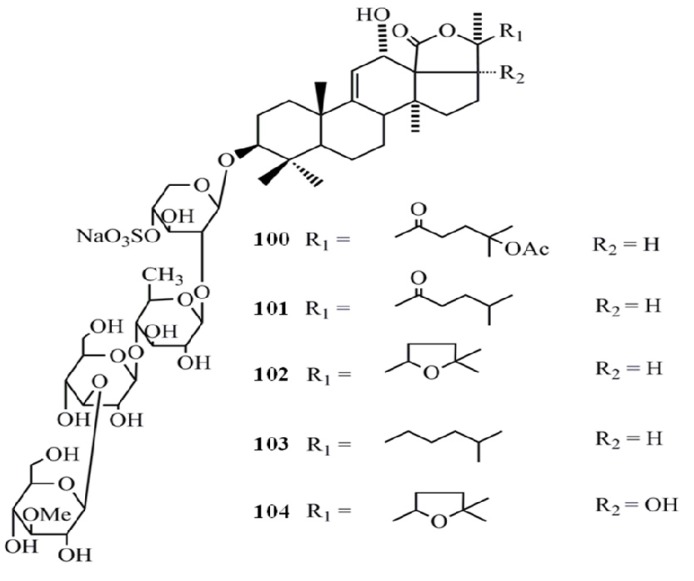
Triterpenoidglycosides fuscocinerosides, pervicoside C and holothurin Afrom sea cucumbers.

Triterpenoid glycosides (saponins) are the major and most abundant type of compounds isolated from sea cucumbers. Saponins are generally perceived as highly active natural product and the sea cucumber saponins have been well characterized for their anti-cancer activities. The cytotoxicity of five triterpene glycosides, fuscocineroside A (**100**), B (**101**), and C (**102**), pervicoside C (**103**) and holothurin A (**104**) isolated from *Holothuria fuscocinerea* Jaeger on human leukemia HL-60 and human hepatoma BEL-7402 cells was analyzed and all compounds have shown a potent cytotoxicity towards both cell lines. However, fuscocineroside C was found to be the most potent (IC_50_ = 0.88, IC_50_ = 0.58 µg/mL) in HL-60 and BEL-7402 cell lines respectively [41] (Figure 7).

**Figure 8:**
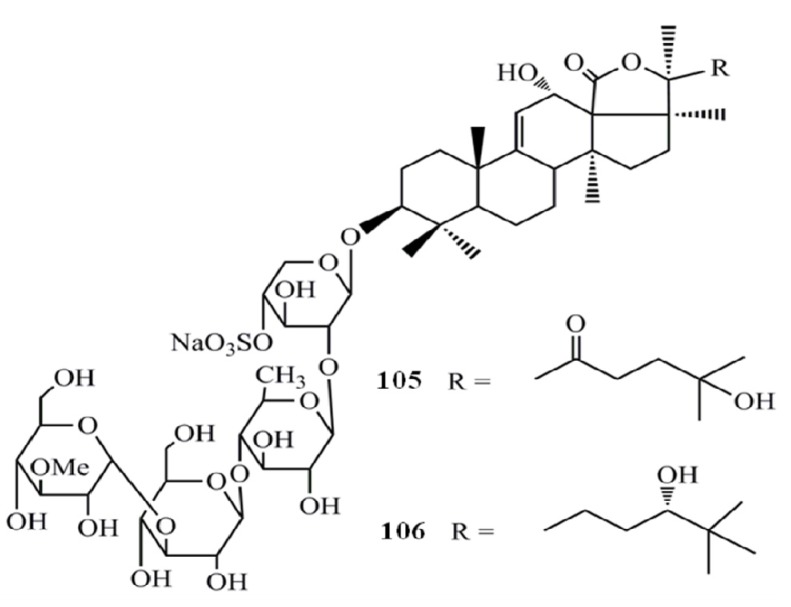
Triterpenoid glycosides holothurin A3 and A4 from sea cucumbers.

The triterpene glycosides from the sea cucumber *Holothuria scabra*, namely holothurin A3 (**105**) and A4 (**106**) found to be strongly cytotoxic to cancer cell lines; human epidermoid carcinoma (KB) and human hepatocellular carcinoma (Hep-G2), with IC_50_ values of 0.87 and 0.32 µg/mL (for compound 7) and of 1.12 and 0.57 µg/mL (for compound 8), respectively [42] (Figure 8).

**Figure 9:**
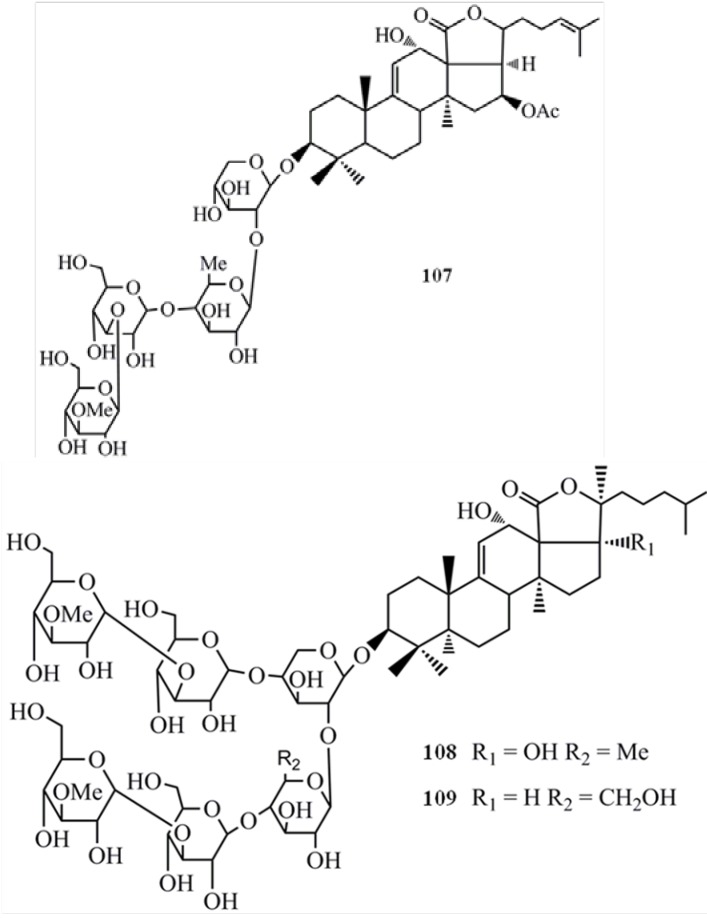
Triterpenoid glycosides arguside A, B and C from sea cucumbers.

**Figure 10:**
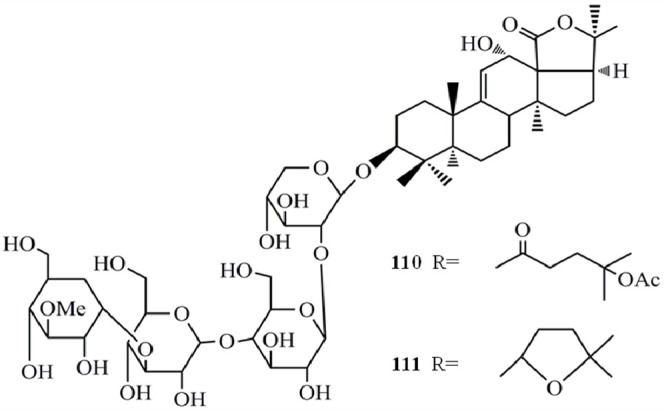
Triterpenoid glycosides arguside D and E from sea cucumbers.

Arguside A (**107**) also exhibited significant cytotoxicity against different human tumor cell lines while showing the highest activity towards human colorectal carcinoma (HCT-116) cells (IC_50_ = 0.14 µM) with more potency than the employed positive control, 10-hydroxycamptothecin (IC_50_ = 0.84 µM) [43]. Argusides B (**108**) and C (**109**) have also shown potent cytotoxicity against human tumor cell lines, adenocarcinomic human alveolar basal epithelial cells (A549), HCT-116, HepG2, and human breast adenocarcinoma (MCF-7) cell lines. The cytotoxicity of the compounds on A549 (**108**-IC_50_ = 0.48 µg/mL, **109**-IC_50_ = 0.43 µg/mL) and HCT-116 (**108**-IC_50_ = 0.46 µg/mL, **109**-IC_50_ = 0.38 µg/mL) cells were more potent than the positive control V-16 (Figure 9). However, there was no significant difference between the cytotoxicity of two compounds [44]. Besides, argusides D (**110**) and E (**111**) have also been tested for their anticancer activities in above human cancer cell lines and revealed a significant activity with IC_50_ values in the range of 3.36–7.77 µg/mL [45] (Figure 10). This finding shows that compounds **108** and **109** are potent cytotoxic agents compared to compounds **110** and **111**. It has been reported that the length and type of sugar moieties of glycosides play an important role in terms of cytotoxic activity against tumor cells and this observation clearly indicates that.

Moreover, the *in vitro* cytotoxicity of impatienside A (**112**) and bivittoside D (**113**) were evaluated extensively by employing seven human cancer cell lines and the results showed that both glycosides exhibited *in vitro* cytotoxicities similar to or better than that of the potent anticancer drug etoposide (V-16) in four human tumor cells, A549 (**112**-IC_50_ = 0.35 µg/mL, **113**-IC_50_ = 0.52 µg/mL), HCT-116 (**112**-IC_50_ = 0.45 µg/mL, **113**-IC_50_ = 0.37 µg/mL), DU-145 (**112**-IC_50_ = 1.14 µg/mL, **113**-IC_50_ = 0.937 µg/mL), KB (**112**-IC_50_ = 1.6 µg/mL, **113**-IC_50_ = 1.42 µg/mL). The structural differences between glycosides **112** and **113** limited to their holostane skeleton, and no significant difference in the cytotoxicity of the two glycosides was found. However, pervicoside C (**103**), an analogue of **113** having the same aglycone but a different sugar chain, isolated from *Holothuria fuscocinerea* Jaeger, exhibited weak activities against HCT-116 and A549 cancer cells, with IC_50_ values of 18.7 and 28.6 µg/mL, respectively [46]. According to these results it is again confirmed that the length and type of sugar moieties of such glycosides play an important role in terms of cytotoxic activity against tumor cells.

**Figure 11:**
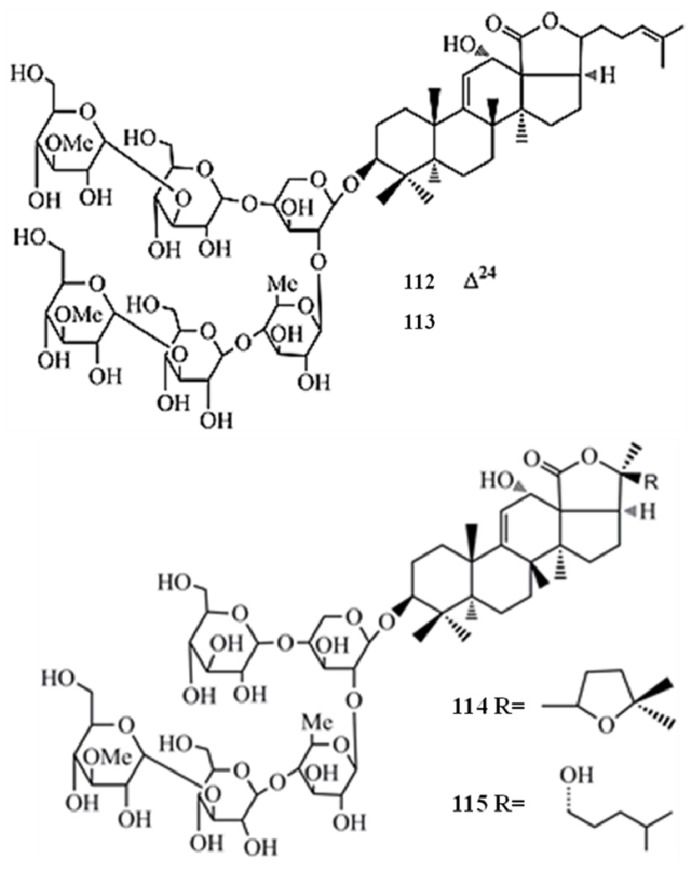
Triterpenoidglycosides impatienside A, bivittoside D, 17-dehydroxyholothurinoside A and griseaside A from sea cucumbers.

17-Dehydroxyholothurinoside A (**114**) and griseaside A (**115**) are identified as promising anticancer agents due to their significantly higher cytotoxicity against four human tumor cell lines, A549 (**114**-IC_50_ = 0.886 µM, **115**-IC_50_ = 1.07 µM) , HL-60 (**114**-IC_50_ = 0.245 µM, **115**-IC_50_ = 0.427 µM), BEL-7402 (**114**-IC_50_ = 0.97 µM, **115**-IC_50_ = 1.114 µM), and human acute lymphoblastic leukemia cell line (Molt-4) (**114**-IC_50_ = 0.34 µM, **115**-IC_50_ = 0.521 µM) compared to the positive control HCP (A549 IC_50_ = 2.35 µM, BEL-7402 IC_50_ = 2.6 µM, HL-60 IC_50_ = 1.9 µM, Molt-4 IC_50_ = 2.2 µM) [47] (Figure 11).

**Figure 12:**
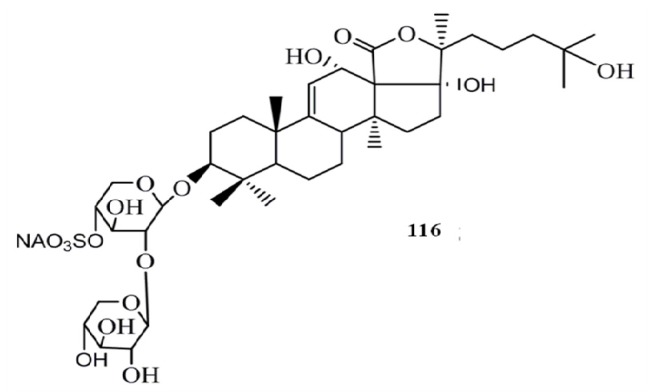
Triterpenoid glycoside hillaside C from sea cucumber.

**Figure 13:**
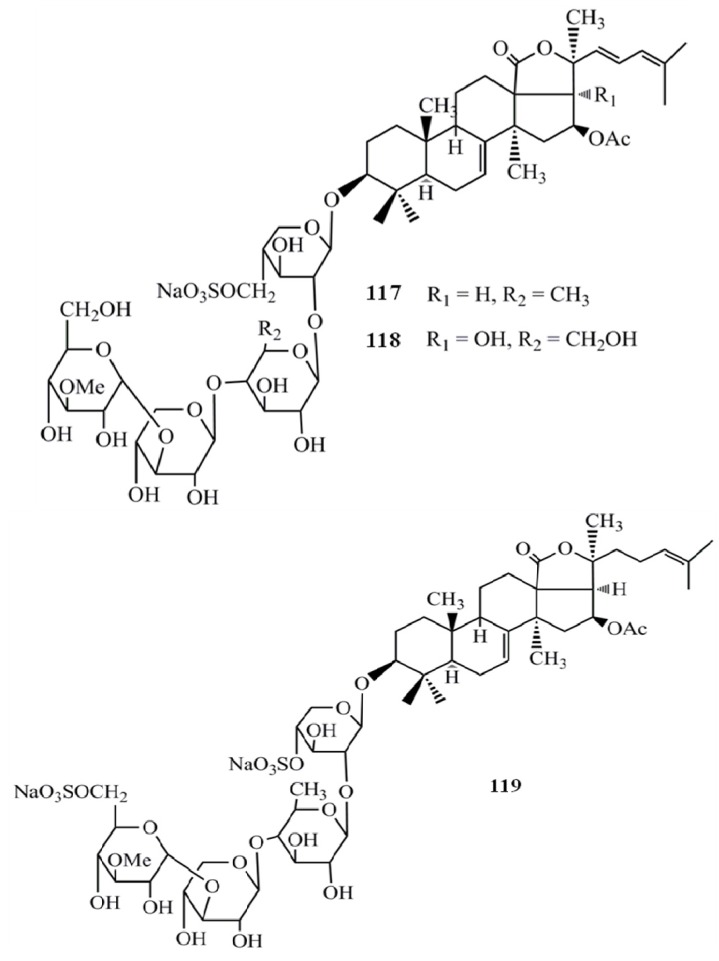
Triterpenoid glycosides intercedensides A, B, and C from sea cucumbers.

Hillaside C (**116**) has also been tested for its anticancer potential against eight human tumor cell lines (A-549, MCF-7, human lung carcinoma cells-IA9, human clear cell carcinoma cells—CAKI-1, human prostate cancer cells—PC-3, KB, KB-VIN, and human colorectal sdenocarcinoma cells-HCT-8) and has exhibited cytotoxicity with IC_50_ values in the range of 0.15–3.20 µg/mL [48] (Figure 12). Compared to the positive control HCP the compound **116** has shown more potent cytotoxicity towards CAKI-1 (IC_50_ = 0.15 µg/mL) and KB-VIN (IC_50_ = 2.81 µg/mL) cell lines. Three new triterpene glycosides, intercedensides A (**117**), B (**118**), and C (**119**) from *Mensamaria intercedens* Lampert, were widely studied for their anticancer activity employing 10 human tumor cell lines (A549, MCF-7, IA9, CAKI-1, human glioblastoma cells—U-87-MG, PC-3, KB, KB-VIN, human skin melanoma cells—SK-MEL-2, HCT-8). Interestingly all compounds showed a significant cytotoxicity against all tumor cell lines within the IC_50_ value range of 0.7–4 µg/mL, and the compounds **117** and **119** showed similar potencies, while compound **118** was generally more potent in all cell lines. Furthermore, compound **117** also exhibited significant *in vivo* antineoplastic activity against mouse Lewis lung cancer and mouse S180 sarcoma, with 48.39% and 57.48% tumor reduction levels [49] (Figure 13).

**Figure 14:**
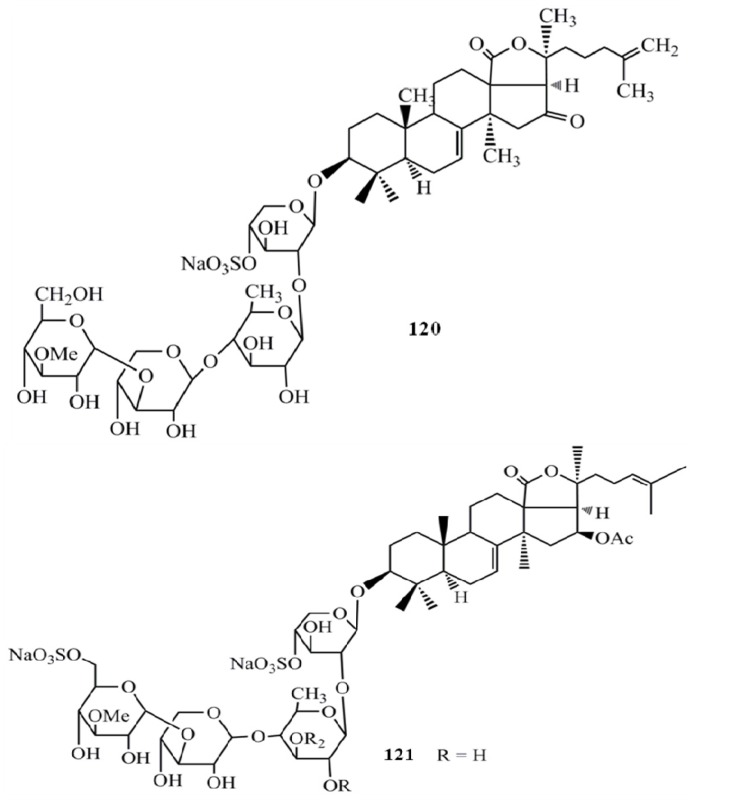
Triterpenoid glycosides philinopside E and intercedenside B from sea cucumbers.

A new sulfated triterpene glycoside from *Pentacta quadrangularis*, philinopside E (**120**) showed a significant cytotoxicity (IC_50_ = 0.75–3.50 µg/mL) against ten tumor cell lines (mouse lymphocytic leukemia cells-P388, HL60, A549, lung adenocarcinoma cells-SPC-A4, gastric carcinoma cells - MKN28, gastric carcinoma cells-SGC7901, BEL7402, human ovarian carcinoma - HO8901, human fetal lung fibroblasts-W138, human epithelial carcinoma cells-A431) [41]. Furthermore, sulfated triterpene glycoside intercedenside B (**121**) from *Pseudocolochirus violaceus* exhibited significant cytotoxicity against cancer cell lines MKN-45 (human gastric adenocarcinoma) and HCT-116 with IC_50_ values in the range of 0.052–0.442 µM and both compounds showed significantly higher activity against HCT-116 compared to the positive control HCP [50] (Figure 14).

**Figure 15:**
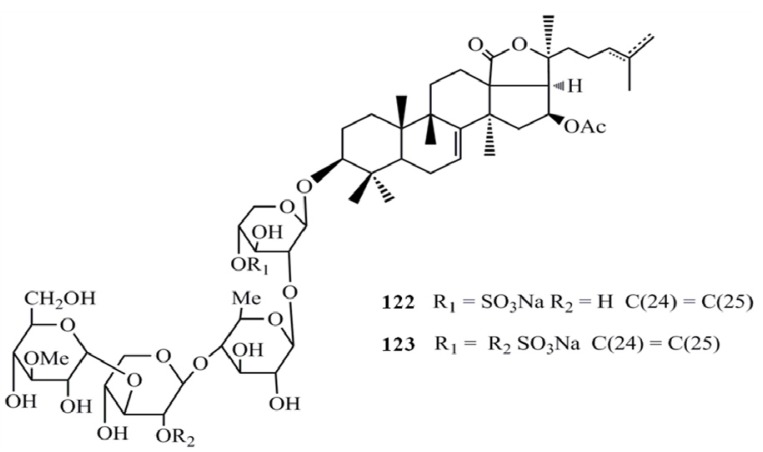
Triterpenoid glycosides philinopsides A and B from sea cucumbers.

Moreover, the sulfated triterpene glycosides, philinopsides A (**122**) and B (**123**) showed significant cytotoxicity (IC_50_ = 0.75–3.50 µg/mL) against ten tumor cell lines (CAKI, HOS, KB-VIN, KB, SM-MEL-2, U87-MG, HCT-8, IA9, A549, and PC3) [51] (Figure 15).

Collectively, all these tripterpene glycosides of sea cucumber are very potent cytotoxic agents towards a wide array of cancer types and the structural properties such as the composition of the sugar moiety and the sulfation in the glycon unit are affecting directly to their cytotoxic potential.

**Figure 16:**
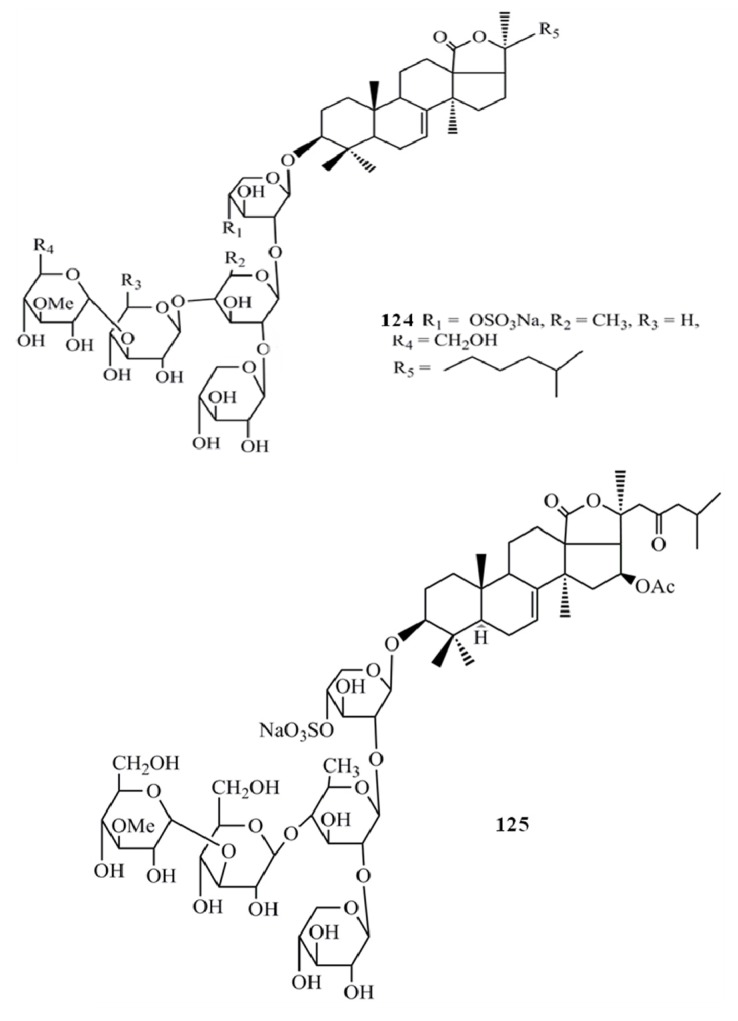
Triterpenoid glycosides frondoside A, cucumarioside A2-2 from sea cucumbers.

**Figure 17:**
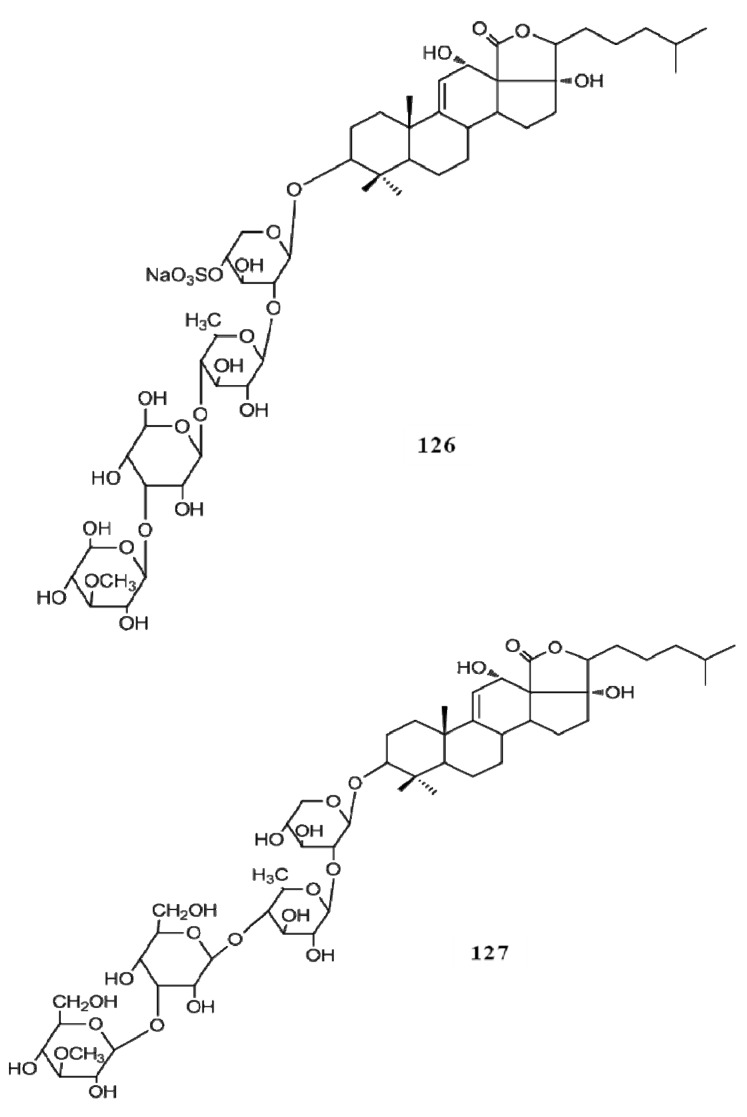
Triterpenoid glycosides echinoside A and ds-echinoside A from sea cucumbers.

Even though a number of saponin compounds have been isolated and identified as potent cytotoxic agents only few of them have been studied to unravel the mode of their cytotoxicity. Among them detailed cytotoxic mechanisms of frondoside A (**124**), cucumarioside A2-2 (**125**), echinoside A (**126**) and ds-echinoside A (**127**) have been reported against several cancer types *in vitro* and *in vivo* (Figure 16, Figure 17). All four compounds have shown their cytotoxicity towards cancer cells by arresting the cell cycle progression via activating the apoptosis pathways which leads to the cell death. Frondoside A has shown potent apoptotic inducing properties against breast cancer, pancreatic cancer and leukemia, cucumarioside A2-2 has studies against leukemia and echinoside A and ds-echinoside A has been characterized against lever cancer [52,53,54]. These compounds activate the intrinsic apoptotic pathway via suppressing the tumor suppressor gene p53. With the suppression of p53, apoptosis pathways are induced and the caspases 3, 7, 8 and 9, the enzymes regulate the cell death process are activated. Interestingly *in vivo* studies have confirmed that frondoside A (100 µg/kg/day) effectively decreased the growth of breast cancer xenografts in athymic mice without exerting any side-effects [52]. Moreover, frondoside A is also capable of inhibiting the cancer cell migration and invasion which will ultimately reduce the progression of cancer to the other parts of the body. Similarly echinoside A and ds-echinoside A treatment (2.5 mg kg^−1^) to the mice bearing H22 hepatocarcinoma tumors has reduced the tumor weight by 49.8% and 55% respectively [54]. These studies evidently prove the higher potential of these compounds as novel natural pharmacological agents against tumor growth and cancer progression.

## 4. Triterpenoids from Marine Algae

Two cytotoxic squalenoid-derived triterpenoids, laurenmariannol (**128**) and (21a)-21-hydroxythyrsiferol (**129**) were isolated and identified from the marine red alga *Laurencia mariannensis*, which was collected off the coast of Hainan and Weizhou Islands of China. Both compounds have displayed significant cytotoxic activity against P-388 tumor cells with IC_50_ values of 0.6 and 6.6 mg/mL, respectively [55].

The red seaweed *Laurencia viridis* is a rich source of squalene derived secondary metabolites. Three squalene-derived brominated triterpenes, dehydrothyrsiferol (**130**), isodehydrothyrsiferol (**131**) and 10-epidehydrothyrisiferol (**132**), isolated from *Laurencia viridis*, have shown potent cytotoxic activities against a number of cancer cell lines [56,57].

Polyethers, iubol (**133**), 22-hydroxy-15(28)-dehydrovenustatriol (**134**), 1,2-dehydropseudo-dehydrothyrsiferol (**135**), and secodehydrothyrsiferol (**136**) exhibited significant cytotoxic activity against a panel of cancer cell lines [58]. Two compounds, 16-hydroxydehydrothyrsiferol (**137**), thyresenol A (**138**) and thyrsenol B (**139**) were also isolated from *Laurencia viridis*, and these compounds have exhibited significant inhibitory action on protein phosphatase at a concentration of 10 mM. Moreover, they have shown potent cytotoxic activity against P388 cell line [59,60].

**Figure 18:**
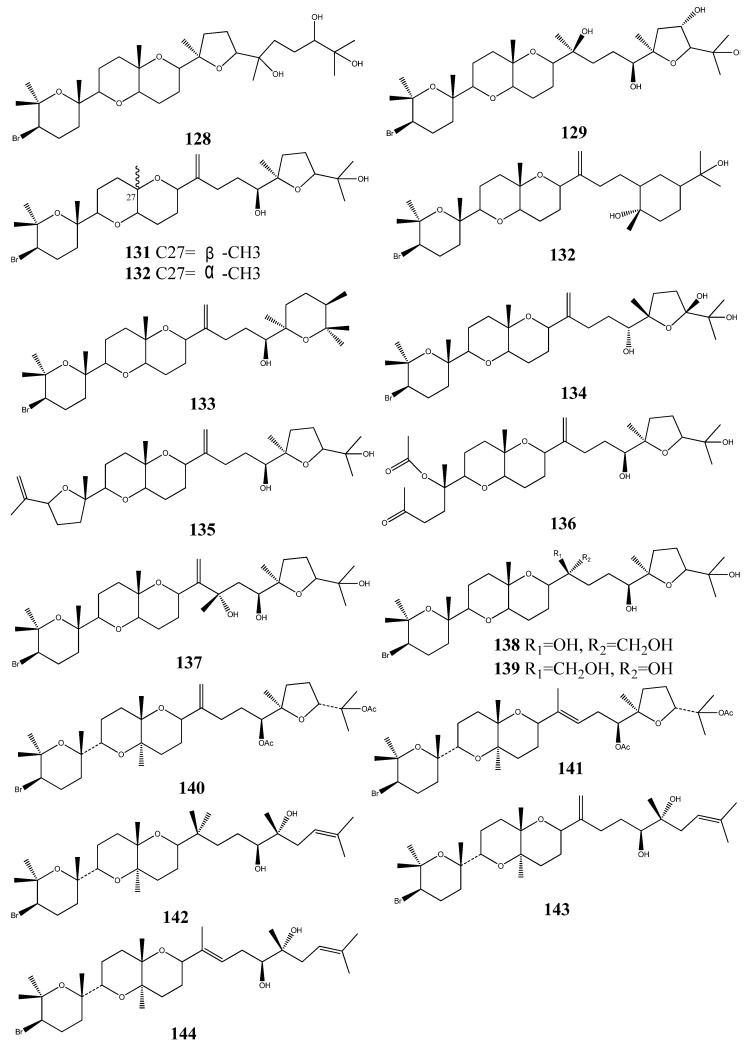
Squalenoid-derived triterpenoids from marine algae.

Five cytotoxic triterpenoids 28-anhy-drothyrsiferyl diacetate (**140**), l5-anhy-drothyrsiferyl diacetate diacetate (**141**), magireol-A (**142**), magireol B (**143**) and magireol C (**144**) were isolated from Japanese red alga *Laurencia obtuse* [61,62] (Figure 18).

**Figure 19:**
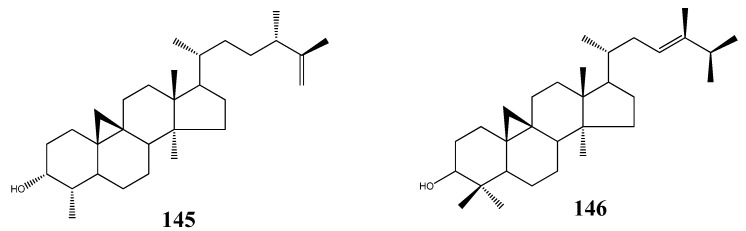
Cycloartane-type triterpenoids from marine algae.

Two cycloartane-type triterpenoids, 3-epicyclomusalenol (**145**), and cyclosadol (**146**) were isolated from brown algae *Kjellmaniella crassifolia*, two compounds were obtained from this species for the first time. 3-epicyclomusalenol (**145**), and cyclosadol (**146**) have been reported to have moderate chemo preventive effects [63,64] (Figure 19).

## 5. Triterpenoids from Marine-Derived Fungi

**Figure 20:**
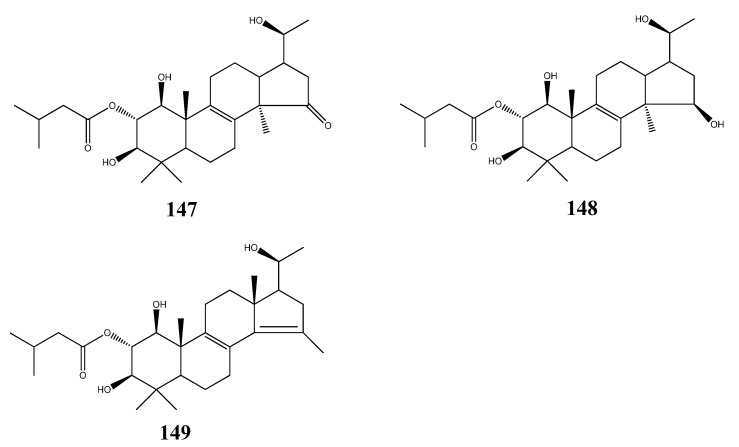
Triterpenoids xylariacins A-C from marine-derived fungi.

Triterpenoids are frequently found in marine source, but have rarely been reported from marine-derived fungi. Rainer Ebel have reviewed 7 triterpenoids from marine-derived fungi [65]. In 2011, three triterpenoids xylariacins A–C (**14**7-1**49**) were isolated from the fermented extract of *Xylarialean sp*. A45, an endophytic fungus of *Annona squamosa* L., and their structures were determined by NMR spectroscopy. These compounds have shown modest cytotoxic activities against human tumor cell line HepG2 [66] (Figure 20).

## 6. Structure Activity Relationships

Even though not much research in this area has been carried out, the anti-cancer activity of triterpenes is believed to be directly correlated to their structural features. As suggested by many authors the bioactivity of the triterpenes is a result of its strong membranolytic activity, and this membranolytic activity is a function of the structural features of the glycosides [67]. In triterpene glycosides the presence of an 18(20)-lactone as the aglycon with at least one oxygen group near it has critical significance for biological activity of glycosides bearing 9(11) double bonds. In the case of glycosides with a 7(8)-double bonds in their aglycon structure, those lacking a 16-keto group are more active than those with a 16-keto group [68]. The characteristics of the attached glycon structure are also critical for the bioactivities of the triterpene glycosides. It has been found that for the actions leading to modification of the cellular membrane, presence of a linear tetrasaccharide chain is significant [69]. And also Maltsev *et al*. [70] have reported that glycosides having quinovose as a second monosaccharide unit are more active over others. The sulfation of the sugar chain is also a significant factor related to bioactivity. A sulfate group at C-4 of the first xylose residue increases the effect against membranes. The absence of a sulfate group at C-4 of the xylose residue in biocides decreases its activity by more than one fold magnitude. On the other hand the presence of a sulfate at C-4 of the first xylose in branched pentanosides having 3-O-methyl groups as a terminal monosaccharide increases activity. However, the same sulfate can decrease the activity of branched pentanosides which have glucose as the terminal residue. Sulfate groups attached to a C-6 position of terminal glucose and 3-O-methylglucose residues impart a great reduction in the activity [67].

## 7. Addressing the Limitations of Using Anti-Cancer Tritepenoids as Therapeutics

Up to date the vast chemodiversity in the oceans has paved the way for natural product chemists to mine for new bioactive compounds. Among them triterpenoids are one of the most studied classes of compounds. Due to the extreme environments in the oceans, survival demands have resulted in the evolution of these sophisticated toxic compounds and this fact is confirmed by the proven toxicity of these compounds in biochemical studies. Triterpenoids derived from sea cucumbers, sponges and algae have been used as ingredients in Traditional Chinese Medicine for years. Even though there are many lead compounds with promising potential to be used as drugs for cancer therapy, the cytotoxicity itself would be a constraint for this purpose, because most of the compounds could be cytotoxic towards normal cells in addition to the cancerous cells. In identification of therapeutics from natural products the preference is given to the compounds having high specificity towards the cancer cells in their cytotoxic action, while minimizing the damage to normal cells [71]. Therefore considerable cytotoxicity studies should be conducted employing the lead compounds before introducing them to the drug development phase. However, advances in drug delivery systems could be applied effectively in specific delivery of therapeutics. Cancer cells carry specific surface receptors that are expressed at higher levels than their normal counterparts. Often these receptors have binding affinity towards specific proteins or peptides [72]. This could be used for the direct targeting of cancer cells which is effectively applicable to develop targeted delivery systems. Nano-drug carriers coated with cancer cell receptor binding factors are a novel and effective approach for the delivery of drugs [73,74]. This method could be used to deliver anti-cancer triterpene glycosides to the cancer tissues and thereby protecting the adjacent normal tissue cells.

Moreover, the possibility of continuous supply of the product and the ecological importance of the triterpene sources such as sponges, sea cucumbers and algae are factors of importance before entering to the drug development phase. Sustainable production of compound through chemical synthesis or culturing of these marine organisms should be ensured. The structural complexities have challenged the chemical synthesis and thus it would limit the entering of these compounds to drug development phase. However, with the advances in synthetic chemistry and understanding of triterpene biosynthetic processes, new opportunities for exploitation of these compounds as drug leads are opening up.
